# The Use of Artificial Neural Networks to Model Selected Strength Parameters of the Giant Miscanthus Stalk

**DOI:** 10.3390/ma19061162

**Published:** 2026-03-16

**Authors:** Sławomir Francik, Tomasz Hebda, Beata Brzychczyk, Renata Francik, Zbigniew Ślipek

**Affiliations:** 1 Department of Mechanical Engineering and Agrophysics, Faculty of Production and Power Engineering, University of Agriculture in Krakow, Al. Mickiewicza 21, 31-120 Kraków, Poland; tomasz.hebda@urk.edu.pl (T.H.); beata.brzychczyk@urk.edu.pl (B.B.); 2 The Faculty of Medicine and Health Sciences, University of Applied Sciences in Nowy Sącz, Kościuszki 2G, 33-300 Nowy Sącz, Poland; 3 Faculty of Engineering Sciences, University of Applied Sciences in Nowy Sącz, Zamenhofa 1a, 33-300 Nowy Sącz, Poland; zslipek@ans-ns.edu.pl

**Keywords:** artificial intelligence, ANN model, carbonaceous materials, biomass, miscanthus, cutting force, cutting work, stalk, stem

## Abstract

The aim of this work was to develop a model using Artificial Neural Networks (ANN) to predict stem cutting parameters for giant miscanthus. Experimental studies were conducted to determine biometric traits: maximum stem diameter (*Dmax*), minimum stem diameter (*Dmin*), stem wall thickness (*THwall*), and strength parameters (cutting force, cutting work) for two giant miscanthus genotypes, depending on the internode number (*NrNod*) and water content (*MC*). A total of 600 measurement results were obtained, which were randomly divided into training (60%), test (20%), and validation (20%) subsets. Two semantic models were adopted: one for predicting stem cutting force (ann1) and one for predicting cutting work (ann2). The independent variables (ANN inputs) were: *Gen*, *MC*, *NrNod*, *Dmax*, *Dmin*, and *THwall*. The ANN creation process was performed using Statistica Neural Networks. For each of the two semantic models (ANN1 and ANN2), 100 neural networks were developed, with the top 10 ANNs retained for further analysis. The criterion for selecting the best neural network was the root mean square error (RMSE) for the test subset. For ANN1, the RMSE values varied from 6.89 N to 8.70 N. For ANN2, the RMSE values varied from 0.086 J to 0.102 J. For the most accurate ANN1-03 (MLP 7-10-1), used to predict grass cutting force, the RMSE values were 6.46 N–6.89 N–4.70 N for the training, test, and validation subsets. For the most accurate ANN2-02 (MLP 7-10-1), used to predict grass cutting work, the RMSE values were 0.0646 J–0.0857 J–0.0596 J for the training, test, and validation subsets.

## 1. Introduction

Carbon materials have been and continue to be an important research area. Research is conducted on the production and modification of carbon materials, the development of new composite materials, and their use in various areas (industrial and environmental) [1,2,3,4,5,6,7]. As a result of this research, various forms of graphite have been developed [5,8].

An example of the use of graphene is the research of Xiao et al. [9]. Taking inspiration from the amorphous/crystalline feature of the heterophase within nacreous platelets, they developed a multilayer heterogeneous pyrocarbon (MHPC) material using chemical vapor deposition (CVD). And then, on this basis, they created carbon fiber-reinforced multilayer heterogeneous pyrocarbon matrix composites (Cf/PHCP). The composite is characterized by high average shielding effectiveness against electromagnetic interference.

The wide application area of carbon materials is due to their unique physical and chemical properties, such as large surface area, highly porous structure, good adsorption properties and high electrical conductivity [5,7,8,10].

Carbon materials are used, among others

- for the production of composites based on high-strength carbon fibers used in space structures and aircraft, rocket engine nozzles, missile cores, thermal shields, thermal insulators and radiators, as well as in sports equipment [2];
- for removing inorganic and organic pollutants using membranes with the addition of activated carbon [4];
- as adsorbents, catalyst carriers, gas storage/separation media [11];
- as electrodes for supercapacitors and materials for electronic applications [10,11];
- as biosensors used in modern analytical chemistry for the detection of biomolecules [12] and in medicine for diagnostics, drug delivery, biomarking and tissue engineering [8].

Industrial and economic development has led to an increasing demand for carbon materials. Traditional carbon materials are obtained from non-renewable sources and fossil fuels. Recently, there has been increasing scientific interest in renewable carbon materials derived from biomass, agricultural residues [6,10], woody biomass [13], marine plant fibers [14], garden waste, and municipal solid waste [15].

Rice husk can be a cheap precursor for the production of carbon-based and hybrid materials for various applications [16], and waste paper sludge and wool have been used for the production of biocomposites [17].

When biomass is heated in the absence or limited presence of oxygen, it produces a porous carbonaceous material—biochar. Biochar consists primarily of highly ordered turbostratically crystalline regions, accompanied by some random amorphous regions [17]. Various thermochemical methods can be used to produce carbonaceous materials from biomass [18,19,20,21]. According to Gallego-Ramírez et al., thermal decomposition of biomass through processes such as carbonization, pyrolysis, torrefaction, and gasification produces a carbon-rich product called biochar [22]. Gutiérrez and Perez, on the other hand, indicate that biochar can be produced not only through gasification, pyrolysis, or combustion for energy recovery, but also during composting and fermentation [15]. Li et al. [23] show that pyrolysis and gasification of tobacco waste allows obtaining valuable products: biochar, tar and syngas.

Biochar is used not only as a fuel, but also in agriculture, construction, and environmental protection [19,21,24]. Biochar is used for soil improvement and land reclamation, energy storage, catalysis, and electrochemistry. It also serves as a precursor in the production of highly porous activated biochar, particularly in processes where adsorption plays a key role (e.g., water treatment) [25,26,27,28].

Scientific research focuses primarily on the use of lignocellulosic biomass, which consists primarily of cellulose, hemicelluloses, and lignin. It is a source of both energy and carbon [26,28]. The percentage of cellulose, hemicelluloses, and lignin in a given type of biomass can vary, significantly affecting its parameters [28,29]. Lignocellulosic biomass can be used to produce polymer composites reinforced with natural fibers [30,31,32], e.g., wood-polymer composites used in the automotive and construction industries, as well as in the packaging and transportation industries [25], to produce “smart” materials, biodegradable polymers for packaging [33], magnetic paper, barrier films, conductive materials, biomembranes [34], and sustainable hydrogen production [35].

Giant miscanthus (*Miscanthus* × *giganteus*) is one of the most productive plant species. Average yields of miscanthus vary from 5 to 55 Mg ha^−1^ [28]. Thanks to its rapid and abundant biomass production it can provide the required amounts of raw material [27,28,36,37,38,39,40]. This plant can be successfully used in the production of compact solid biofuels [41,42,43,44], biogas, and bioethanol [45,46,47]. Miscanthus biomass has also been used in the production of technical papers, cardboard, packaging plywood, and fibers for packaging lining. It can also be a substitute for plastics in packaging production [48]. Giant miscanthus can also be a source of cellulose nanocrystals for PVAc (polyvinyl acetate) nanocomposites [49]. Huber et al. investigated the effect of particle size of miscanthus biochar on a polyamide polymer [50]. Bartoli et al. tested the mechanical properties of an epoxy-based composite with commercial miscanthus biochar [51].

When designing machines, it is important to know their physical, strength, mechanical, and aerodynamic properties [52]. Determining the mechanical properties of plant-derived materials is essential for designing dedicated processing machinery and selecting optimal operating parameters. The mechanical properties of these types of materials vary significantly depending on environmental conditions (e.g., humidity), making the process difficult. The results of research on the biomechanical properties of industrial plant stems provide valuable data for the design of reliable and more efficient biomass processing equipment [28,53,54,55,56,57,58,59,60,61,62,63,64,65].

Typical laboratory tests to determine the biomechanical properties of plant stems include: bending tests, compression tests along or across the grain; tensile tests; shearing tests; cutting tests, and impact strength tests [28,58,59,60,61,62,65,66,67,68,69].

Research on the process of cutting plants is important in the context of harvesting machines (designing various types of cutting units) and shredding of stalks, as well as reducing the energy consumption of these processes [55,56,57,60,70,71,72]. Many research teams are investigating the energy requirements for cutting various crops, such as winter rapeseed, wheat, triticale, hemp, peas, rice, soybean stalks, and pyrethrum flowers [73,74].

Studies have also been conducted on the cutting process of miscanthus. Maughan et al. [73] conducted a study on the amount of energy consumed during cutting during miscanthus harvesting (they examined the effect of cutting speed, blade angle, and blade mounting on energy consumption).

A team of researchers from the University of Bucharest, POLITEHNICA, has been conducting research on the cutting process of miscanthus stalks for many years. They conducted studies on the cutting process of miscanthus stalks using V-shaped cutting blades, in which they determined the cutting resistance (cutting force and cutting energy) for different internodes [57,75]. Moiceanu et al. studied [60] the mechanical behavior of giant miscanthus (*Miscanthus* × *giganteus*) stems when cut with an oblique blade at different rake angles. They also simulated the mowing operation of giant miscanthus stems using FEM (finite element method) [76].

Toleu and Liu [72] conducted impact tests on giant miscanthus stems using an impact tester. The tests were performed at nodes and internodes. The cutting force, sample diameter, and cutting speed were recorded. The specific cutting force and energy were then calculated.

The next stage of scientific research, after performing experimental measurements, is usually an attempt to describe phenomena using various types of mathematical models (e.g., regression models). One relatively new tool for mathematical modeling is Artificial Neural Networks (ANN). Examples of the use of ANNs for modeling various phenomena and processes have shown that they are characterized by very high accuracy compared to traditional mathematical models [40,77,78,79,80,81,82,83].

ANNs have been successfully used in many areas of science, for example in bioinformatics, biochemistry, medicine, meteorology, economics, robotics, food security, climatology, agrophysics and agricultural engineering [40,41,78,79,81,82,83,84,85,86,87,88,89,90].

Neural networks have also been used to model the cutting process of stalks and stem. Mahdavian et al. [73] developed an ANN to predict the shear energy of rapeseed stalks depending on the plant variety, stalk moisture content, loading rate, knife type, and fertilizer application. Azadbakht et al. [52] developed five neural networks (feedforward networks) to predict various mechanical properties of rapeseed stalks: cutting force, cutting energy, cutting work, cutting power, and shearing strain. They used the stem diameter, shear rate, and blade angle as inputs to the ANNs (independent variables).

Elwakeel et al. [91] used Deep Neural Networks and Feedforward Neural Networks to evaluate the performance and predict the optimal operating conditions of a sugarcane stalk harvester. The ANNs used cutting height, number of knives, row spacing, average stalk diameter, forward speed, knife rotational speed, knife linear speed, and speed ratio as inputs. The outputs were machine performance, number of uncut stalks, cutting efficiency, field capacity, throughput capacity, fuel consumption, total operating costs (USD/h), total operating costs (USD/ha), and power requirements.

ANN models allow for the precise quantitative description of various processes. They are characterized by greater accuracy than other types of mathematical models. With experimental results, computationally efficient ANNs can be created without knowledge of the physical relationships between the dependent and independent variables. Such models are therefore an excellent tool for conducting simulation processes. ANNs allow for determining the strength parameters of plant materials based solely on their geometric features—which are easy to measure. This allows for the avoidance of tedious strength tests [40].

No neural models have yet been developed to describe the process of static cutting of miscanthus stems. Therefore, the aim of this work was to develop models using Artificial Neural Networks (ANNs) to predict cutting parameters of giant miscanthus stems.

## 2. Materials and Methods

To achieve the intended aim of this work, it was necessary to obtain experimental data—the results of laboratory tests on the process of cutting miscanthus stems. This data was then used to develop ANN models.

### 2.1. Gathering Experimental Data—Static Cutting Test Results

Miscanthus stalks (plant material) were collected in February and March on experimental plots of the Experimental Station in Puławy Osinach, Institute of Soil Science and Plant Cultivation State Research Institute, Puławy, Poland.

Stalks of two miscanthus genotypes were collected: *Miscanthus* × *giganteus* (M.Gig) and genotype M.117.

**Figure 1:**
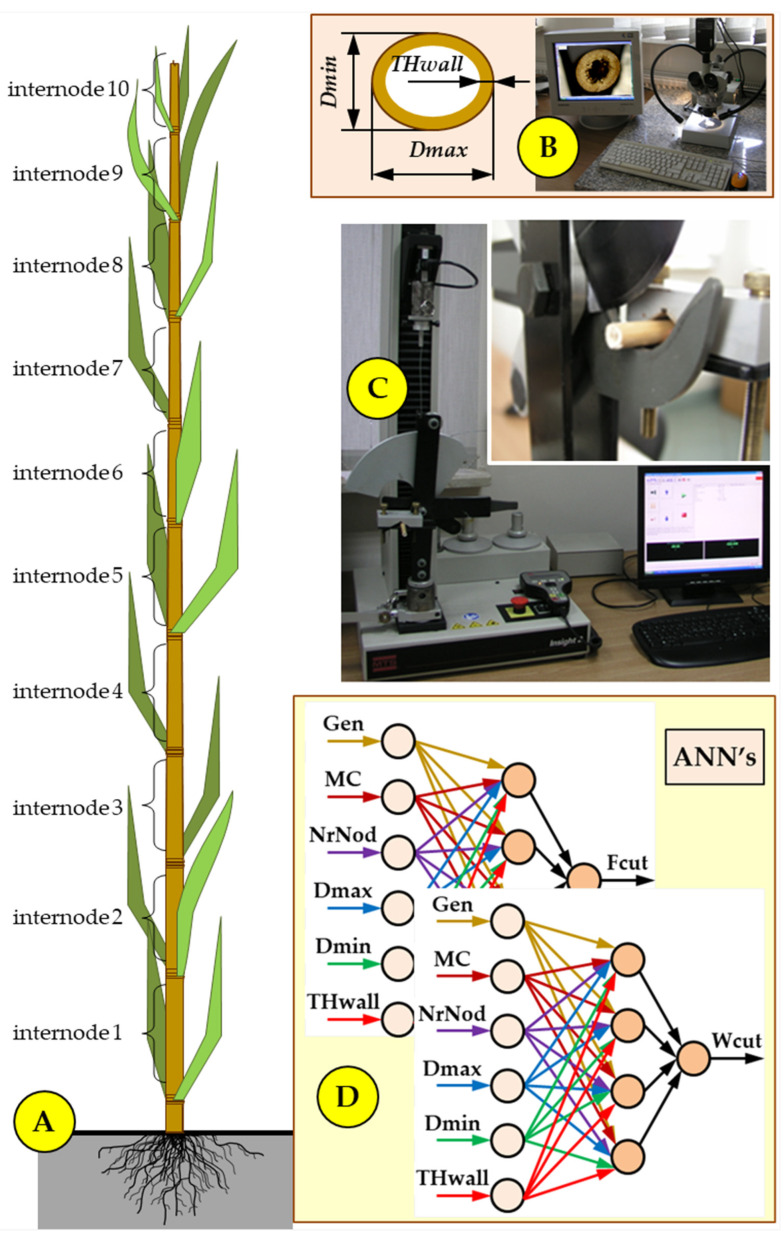
Schematic diagram of the experimental methodology: (**A**) Representation of the miscanthus stem and internodes used for sample preparation; (**B**) measurement setup for determining the characteristic dimensions of samples: *Dmin*—minimum stalk diameter, *Dmax*—maximum stalk diameter and *THwall*—stalk wall thickness; (**C**) a scheme of a static cutting test; (**D**) development of artificial neural networks [40].

Samples were taken from randomly selected miscanthus stems (devoid of leaves) from individual internodes (*NrNod* from 1 to 10)—Figure 1A.

The geometric dimensions of the miscanthus stem cross-section (minimum stalk diameter *Dmin*, maximum stalk diameter *Dmax*, and stalk wall thickness *THwall*—Figure 1B) were determined using a microscope with a digital camera. The captured images were used to measure the cross-sectional dimensions of the stems using the Multiscan program (Computer Scanning System, Warsaw, Poland) [28]. The geometric characteristics of miscanthus stems are provided in Table A1 and Table A2 (Appendix A). The geometric characteristics of miscanthus stems are provided in Table A1 and Table A2 (Appendix A).

Moisture content (*MC*) in miscanthus stems was determined using the drying method, independently for each internode [28].

A static cutting test of miscanthus genotypes was conducted using the methodology proposed by Mudryk [55]. A strain gauge head with a measuring range of up to 2 kN was used for the tests (Figure 1C). The strain gauge head travel speed was 25 mm min^−1^.

During the cutting test, the following parameters were recorded: cutting work—Wcut, cutting force—Fcut, time and displacement.

Measurements were taken for 15 stems (replicates) of each of the two miscanthus genotypes, for two moisture content levels, and 10 internodes. A total of 600 results (samples) were obtained.

### 2.2. Development of Neural Models of Static Cutting of Miscanthus Stems

ANN models were created according to the methodology developed and verified in our previous studies [40,92,93].

#### 2.2.1. Semantic Models Formulation

The first step in creating models using artificial neural networks is developing a semantic model. This involves selecting independent variables (network inputs) and a dependent variable or variables (network outputs).

In our research, we used the following as inputs to the neural networks:

- *Gen*—genotype (-);
- *MC*—moisture content (%);
- *NrNod*—internode number (-),
- *Dmax*—maximum stem diameter (mm),
- *Dmin*—minimum stem diameter (mm),
- *THwall*—stem wall thickness (mm).

We used the maximum cutting force (*Fcut*) and the cutting work (*Wcut*) as the ANN’s outputs. We formulated two semantic models:

(1) $$ANN1:\;Fcut=f\left(Gen;MC;NrNod;Dmax;Dmin;THwall\right)$$

(2) $$ANN2:\;Wcut=f\left(Gen;MC;NrNod;Dmax;Dmin;THwall\right),$$

where:

*Fcut*—maximum cutting force (N);

*Wcut*—cutting work (J).

#### 2.2.2. Selecting ANN’s Architecture and Carrying out the Process of Learning

To develop neural models, we used Statistica Neural Networks software (Dell Inc. (2016), Round Rock, TX, USA. Dell Statistica (data analysis software system), version 13, software.dell.com), StatSoft, Inc., Tulsa, OK, USA. In our research, we used multilayer perceptron neural networks (MLP).

To obtain ANNs characterized by minimal error values, we applied a multiple iteration procedure to the neural network training process using the Automatic Designer function. The training process was repeated 100 times for each of the two semantic models (described by Equations (1) and (2)), and the 10 best neural networks were retained. The training process was performed for different numbers of neurons in the hidden layer (from 3 to 11 neurons) and different activation functions in the individual layers (linear, logistic, hyperbolic tangent, exponential) [92,93].

The BFGS (Broyden–Fletcher–Goldfarb–Shanno) algorithm was used for training [40,92].

The experimental measurement results were randomly divided into learning subset (60%), testing subset (20%), and validation subset (20%). The final learning subset consisted of 360 cases, and the testing and validation subsets each had 120 cases.

#### 2.2.3. Choosing and Assessing the Best ANN Models

The selection of the best neural model, among 10 ANNs developed using the Automatic Designer function for each of the two adopted semantic models, was made based on the root mean square error (RMSE) value for the test subset (Equation (3)) [40,92,93,94,95,96]:

(3) $$RMSE=\sqrt{\frac{1}{N}\sum_{i=1}^{N}{\left(Y_{ME,i}-Y_{ANN,i}\right)}^{2}}$$

where:

*RMSE*—root mean-square error (N) or (J),

*Y_ME,i_*—measured value of output (N) or (J),

*Y_ANN,i_*—calculated by ANN value of output (N) or (J),

*N*—number of cases (-).

For the selected best neural models, the mean percentage error (MAPE) values were also calculated [40,92,96,97]:

(4) $$MAPE=\frac{1}{N}\sum_{i=1}^{N}\left|\frac{Y_{ME,i}-Y_{ANN,i}}{Y_{ME,i}}\right|\cdot 100\text{\%}$$

where:

*MAPE*—mean absolute percentage error (%),

*| … |*—absolute value (-).

*Y_ME,i_*—measured value of output (N) or (J),

*Y_ANN,i_*—calculated by ANN value of output (N) or (J),

*N*—number of cases (-).

#### 2.2.4. Sensitivity Analysis for the Best Neural Models

Sensitivity analysis was performed for the two best neural models, which allowed us to determine the importance of individual independent variables (ANN inputs) for model accuracy. The calculated error quotient values allow us to determine which variables are important for model accuracy. The higher the error quotient value, the more important the input is for model accuracy [40,92,93,94,98,99].

## 3. Results

**Figure 2:**
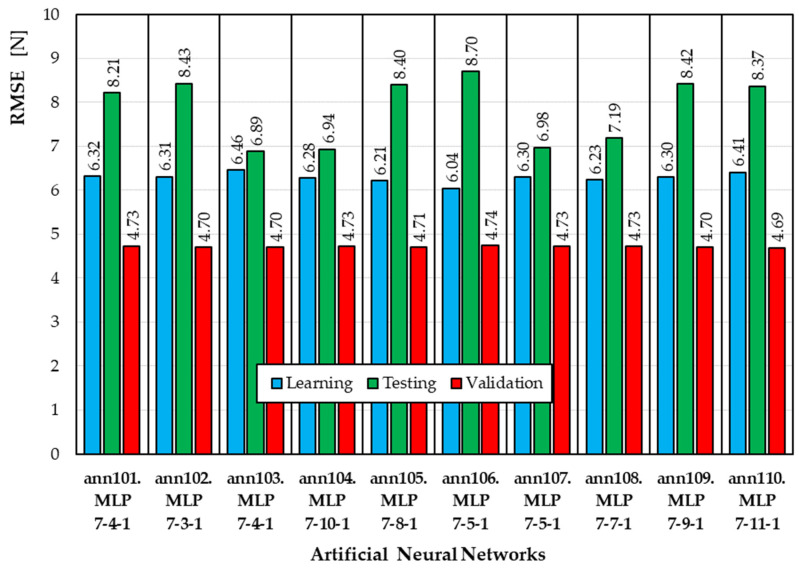
RMSE values for ANNs developed according to the ANN1 semantic model.

Figure 2 summarizes the RMSE values obtained by the top 10 neural networks developed using the ANN1 semantic model (output variable Fcut). For the training subset, the RMSE ranges from 6.04 N to 6.45 N. For the test subset, the RMSE ranges from 6.89 N to 8.70 N. The best model is the ann103 model (MLP 7-10-1), for which the RMSE calculated for the test set is the lowest.

**Figure 3:**
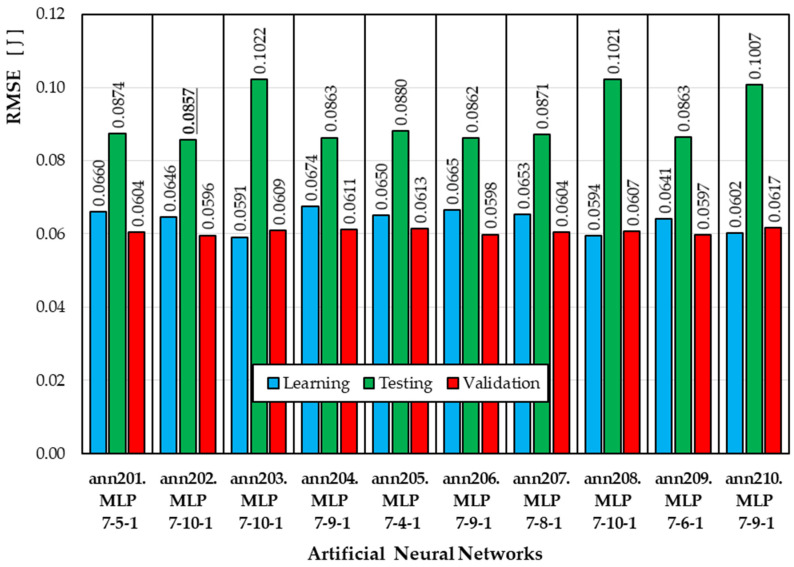
RMSE values for ANNs developed according to the ANN2 semantic model.

Figure 3 summarizes the RMSE values obtained by the top 10 neural networks developed using the ANN2 semantic model (output variable Wcut). For the training subset, the RMSE ranges from 0.0591 J to 0.0674 J. For the test subset, the RMSE ranges from 0.0857 J to 0.1022 J. The best model is the ann202 model (MLP 7-10-1), for which the RMSE value calculated for the test set is the lowest.

**Figure 4:**
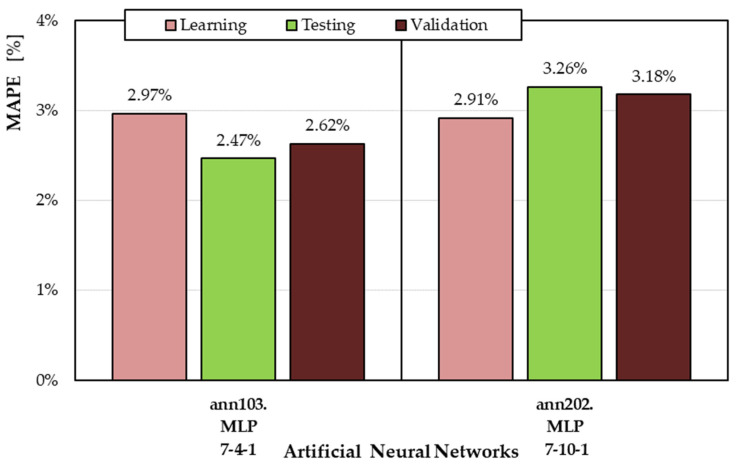
MAPE values for the best ANNs developed according to the ANN1 and ANN2 semantic models.

Figure 4 summarizes the MAPE values for the two selected ANN models across the different subsets (learning, testing, and validation). The MAPE values for both ann103 and ann202 are very low—below 3.5%. The MAPE values for the validation subset do not differ from the MAPE values for the learning and test subsets, indicating that the neural models retain their ability to generalize and that the ANNs were not overfitted.

Table 1 lists the architecture, learning algorithm and activation functions of the selected ANNs.

The results of sensitivity analysis for selected ANNs (ann103 and ann202) are presented in Table 2. Error quotient values (greater than 1) indicate that variables make a significant contribution to the model’s predictive ability. For both models, the most important input variables are stem wall thickness (*THwall*), minimum stem diameter (*Dmin*), and maximum stem diameter (*Dmax*). The least important input variables are genotype (*Gen*) and internode number (*NrNod*). The low error quotient (ranked 4th) for the *MC* variable results from the small difference in moisture content (20.7% and 25.6% in our study). For plant materials, the effect of *MC* on strength parameters is greater with larger differences in moisture content. This obviously limits the applicability of our neural models. However, given that cutting processes (harvesting, chopping) are conducted within such moisture content ranges, this is not a problem.

**Figure 5:**
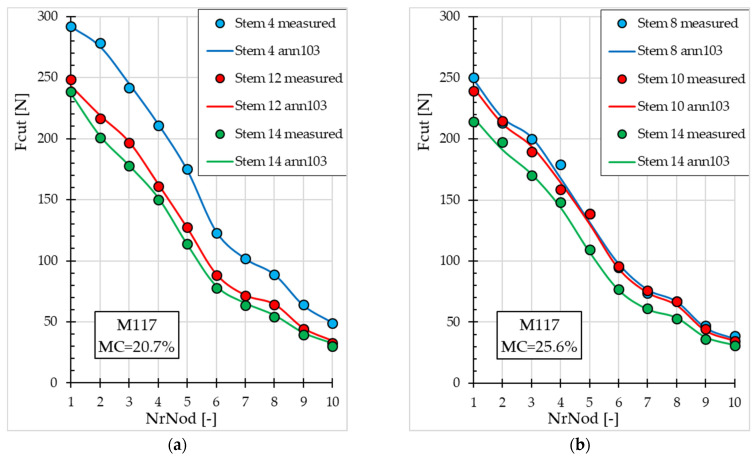
Maximum cutting force values for miscanthus M117 depending on the internode number (validation subset): (**a**) for water content 20.7%; (**b**) for water content 25.6%.

**Figure 6:**
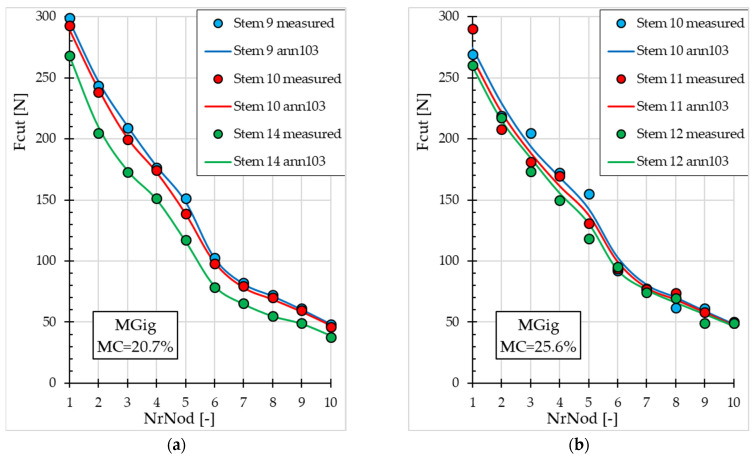
Maximum cutting force values for miscanthus MGig depending on the internode number (for the validation subset): (**a**) for water content 20.7%; (**b**) for water content 25.6%.

Figure 5 and Figure 6 present Fcut values (measured and obtained with ann103) depending on *NrNod* for both miscanthus genotypes at different *MC* values. Graphs are presented for miscanthus stalks from the validation subset that were not used in the ANN training process.

**Figure 7:**
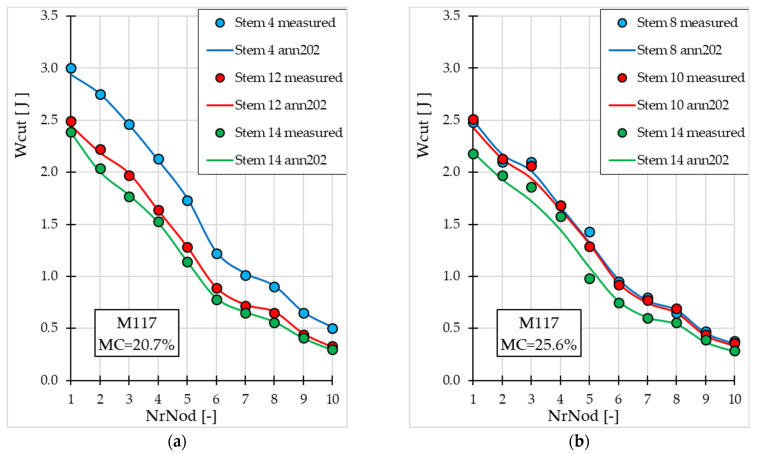
Maximum cutting work values for miscanthus M117 (for the validation subset) depending on the internode number: (**a**) for water content 20.7%; (**b**) for water content 25.6%.

**Figure 8:**
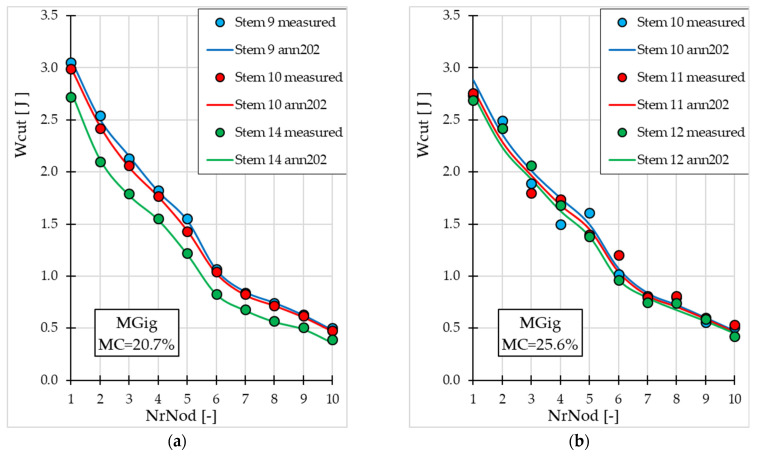
Maximum cutting work values for miscanthus MGig depending on the internode number (for the validation subset): (**a**) for water content 20.7%; (**b**) for water content 25.6%.

Figure 7 and Figure 8 present the Wcut values (measured and obtained from ann202) depending on *NrNod*, for miscanthus stalks from the validation subset. Graphs for both miscanthus genotypes at different *MC* values (20.7% and 25.6%) are presented.

## 4. Discussion

The developed neural models ann103 and ann202 demonstrate good accuracy and generalization ability, as demonstrated by the error values calculated for the validation subset (MAPE = 2.62% for ann103, MAPE = 3.18% for ann202).

These values are comparable to the MAPE values obtained for the ANNs we developed to predict the bending modulus and maximum bending stress of miscanthus stems [40]. The two ANNs for determining the bending modulus achieved MAPEs of 2.3% and 2.2% for the validation subset. The neural models for predicting the maximum bending stress had MAPEs of 4.1% and 0.2%.

Azadbakht et al. [52] developed ANNs to determine canola stem cutting parameters. They used cutting speed, cutting angle, and stem diameter as input variables. The output variables of the neural networks were cutting force, shear strength, cutting energy, cutting power, and cutting work. Their calculated MAE for cutting force was 0.0245, and for cutting work 0.0506. Unfortunately, they did not provide MAPE values.

Mahdavian et al. [73] developed an MLP-type ANN to determine the shear energy of canola stems depending on the plant variety, stem moisture content, type of the cutting knife and the amount of urea fertilizer. The best neural network had 15 neurons in the first hidden layer and 6 neurons in the second hidden layer. The MSE for the training subset was 0.00015.

In the literature, no neural models analogous to the ones we developed, i.e., those allowing for determining cutting parameters for miscanthus stalks, were found. However, ANNs are used to model the cutting processes of plant materials. An example is the study by Elwakeel et al. [91], who used a Feedforward Neural Network and a Deep Neural Network (DNN) to evaluate the performance and predict the optimal operating conditions of a sugarcane stalk harvester. They used cutting height, number of knives, row spacing, average stalk diameter, forward speed, knife rotational speed, linear knife speed, and speed factor as inputs for the ANN. The outputs included machine performance (qualitative assessment), number of uncut stalks, cutting efficiency (%), field capacity (ha/h), throughput capacity (t/h), fuel consumption (l/h), total operating costs (USD/h), total operating costs (USD/ha), and power requirements (kW). Their results showed that DNNs better describe the collection process, achieving lower error values. The RMSE values for DNNs, depending on the output variable, range from 0.0165 to 0.3401.

The maximum cutting force values obtained in our studies varied from 381 N (for *NrNod* = 1) to 24 N (for *NrNod* = 10). This is due to the fact that as the internode number increases, the diameters (*Dmin* and *Dmax*) and the stem wall thickness (*THwall*) decrease.

According to studies by other authors, the average cutting force values for a V-shaped blade with an opening angle of 50° and cutting edge angles from 10° to 50° were in the range of 273.9–585.2 N for the basal internodes and in the range of 113.0–192.9 N for the seventh internode [57].

However, the average cutting force values for the knife with an opening angle of 30° for one internode varied from 502.0 N to 701.3 N (for different angles of the cutting knife edge). For seven internodes, a change from the value of 197.6 N to the value of 385.6 N occurred [75].

These studies confirm our observations that pruning force depends on the number of internodes. Of course, it also depends on the transverse dimensions of the stem, as demonstrated by our ANN models.

In other research, the research team of Moiceanu et al. [60] determined the values of specific cutting force and specific cutting energy in static cutting tests of giant miscanthus stems. Experimental measurements were performed using knives with a cutting angle of 30° and sharpening angles of 10°, 30°, and 50°. The tests were conducted only for the first two internodes. The number of replicates was 10. The average values of the specific cutting force ranged from 31 to 34 N·mm^−1^, for different sharpening angles.

Toleu and Liu [72] studied the dynamic cutting properties of giant miscanthus stems using an Impact Tester. They investigated the effect of cutting blade type, sample support method, and sampling location (top or bottom) on the cutting force and energy of a single plant stem. The cutting force values they obtained ranged from 441 to 469 N·cm^−2^.

The decrease in *Fcut* values with increasing *NrNod* is shown in Figure 5 and Figure 6 (for the validation subset). For miscanthus M117, *Fcut* for *NrNod* = 1 ranges from 238 N to 292 N (for *MC* = 20.7%) and from 214 N to 250 N (for *MC* = 25.6%)—Figure 5. However, for miscanthus MGig, Fcut for *NrNod* = 1 ranges from 268 N to 299 N (for *MC* = 20.7%) and from 260 N to 290 N (for *MC* = 25.6%)—Figure 6. Therefore, the maximum shear force values do not differ significantly. For *NrNod* = 10, Fcut varies from 30 N to 49 N (for *MC* = 20.7%) and from 31 N to 39 N (for *MC* = 25.6%) for miscanthus M117. For miscanthus MGig, Fcut = 37 N–48 N (for *MC* = 20.7%) and from 49 N to 50 N (for *MC* = 25.6%) for *NrNod* = 10.

Analysis of the fit of the Fcut results generated by the ANN to the measured results for both miscanthus M117 (Figure 5) and miscanthus MGig (Figure 6) confirms the good accuracy of the ann103 neural model. The best fit (lowest MAPE value = 0.62%) occurs for blade 4 (M117, *MC* = 20.7%)—Figure 5a. The worst fit occurs for miscanthus MGig (*MC* = 25.6%), blade 10 (MAPE = 5.79%), and blade 12 (MAPE = 5.30%)—Figure 6b.

The cutting work values we obtained varied from 4.02 J (for *NrNod* = 1) to 0.24 J (for *NrNod* = 10). In their publication, Mudryk et al. [55] reported unit cutting work values for giant miscanthus stems of 0.032 J·mm^−2^ for 20% moisture content and 0.027 J·mm^−2^ for 33% moisture content. After converting Wcut to unit cutting work, we obtained values ranging from 0.051 J·mm^−2^ (for *NrNod* = 1) to 0.012 J·mm^−2^ (for *NrNod* = 10).

Also, in the case of cutting work, we observe a decrease in the Wcut value with increasing internode number—Figure 7 and Figure 8. The accuracy and generalization ability of the ann202 model, developed for Wcut prediction, is confirmed by a comparison of experimental values and those generated by the ANN model, both for miscanthus M117 (Figure 7) and for MGig (Figure 8).

## 5. Conclusions

The neural models we developed enable the simulation of static cutting of giant miscanthus stalks. The results of such simulations can be used in the design of machines and devices for the mechanical processing of miscanthus biomass. This is a goal often emphasized in the literature. Mathematical models constitute a typical stage in scientific research. After conducting experimental studies, mathematical models are developed, for example, in the form of regression functions, which summarize the obtained results and provide a synthetic record of the relationships found. Naturally, the goal is to obtain the most accurate models of this type. Because ANNs allow for a very precise description of nonlinear relationships between dependent and independent variables, they are frequently used.

The maximum cutting force values obtained in our studies varied from 381 N (for *NrNod* = 1) to 24 N (for *NrNod* = 10). This is due to the fact that as the internode number increases, the diameters (*Dmin* and *Dmax*) and the stem wall thickness (*THwall*) decrease. The cutting work values we obtained varied from 4.02 J (for *NrNod* = 1) to 0.24 J (for *NrNod* = 10). Also, in the case of cutting work, we observe a decrease in the Wcut value with increasing internode number.

The developed neural models ann103 and ann202 demonstrate good accuracy and generalization ability, as demonstrated by the error values calculated for the validation subset (MAPE = 2.62% for ann103, MAPE = 3.18% for ann202).

## Figures and Tables

**Table 1:** Characteristic of selected neural models.

ANN	Type and Architecture of the Neural Network	Neuron Number in Input Layer	Number of Neurons in Hidden Layer	Number of Neurons in Output Layer	Learning Algorithm	Activation Function in Hidden Layer	Activation Function in Output Layer
ann103	MLP 7-4-1	7	4	1	BFGS ^1^ 52	Exponential	Exponential
ann202	MLP 7-10-1	7	10	1	BFGS 21	Exponential	Linear

^1^ BFGS—Broyden-Fletcher-Goldfarb-Shanno algorithm; MLP—multilayer perceptron.

**Table 2:** Sensitivity analysis for selected neural models, for the all data subset.

ANN Model		Independent Variables (ANN Inputs)					
		*THwall* (mm) *	*Dmin* (mm) *	*Dmax* (mm) *	*MC* (%) *	NrNod *	*Gen* *
ann103	Error quotient	41.2	23.1	13.0	2.8	1.1	1.4
(MLP 7-4-1)	rank	1	2	3	4	6	5
ann202	Error quotient	25.9	23.3	15.6	2.5	2.3	1.2
(MLP 7-10-1)	rank	1	2	3	4	5	6

* *Gen*—genotype (-); *MC*—moisture content (%); *NrNod*—internode number (-); *Dmax*—maximum stem diameter (mm); *Dmin*—minimum stem diameter (mm); *THwall*—stem wall thickness (mm).

## Data Availability

The original contributions presented in this study are included in the article. Further inquiries can be directed to the corresponding authors.

## Appendix A

**Table A1:** Geometric dimensions of the Miscanthus M117 stems.

*MC* [%]	*NrNod*	*Dmax* [mm] Mean (Std. Dev.)	*Dmin* [mm] Mean (Std. Dev.)	*THwall* [mm] Mean (Std. Dev.)
20.7	1	9.25 (0.52)	8.54 (0.07)	1.43 (0.07)
	2	8.84 (0.68)	8.37 (0.16)	1.40 (0.07)
	3	8.50 (0.67)	8.35 (0.28)	1.30 (0.06)
	4	8.29 (0.67)	7.61 (0.34)	1.24 (0.06)
	5	8.16 (0.69)	6.80 (0.43)	1.13 (0.05)
	6	7.23 (0.72)	5.80 (0.47)	1.06 (0.05)
	7	6.86 (0.72)	5.61 (0.50)	0.94 (0.04)
	8	6.70 (0.76)	5.55 (0.52)	0.86 (0.04)
	9	6.38 (0.72)	4.70 (0.49)	0.75 (0.03)
	10	6.30 (0.70)	4.60 (0.46)	0.59 (0.03)
25.6	1	9.29 (0.37)	8.59 (0.11)	1.44 (0.06)
	2	8.96 (0.46)	8.36 (0.16)	1.37 (0.06)
	3	8.45 (0.37)	8.43 (0.29)	1.31 (0.05)
	4	8.24 (0.37)	7.61 (0.29)	1.25 (0.05)
	5	8.10 (0.39)	6.75 (0.34)	1.14 (0.05)
	6	7.18 (0.41)	5.76 (0.35)	1.07 (0.04)
	7	6.83 (0.42)	5.59 (0.34)	0.95 (0.04)
	8	6.64 (0.41)	5.53 (0.34)	0.86 (0.03)
	9	6.30 (0.43)	4.62 (0.30)	0.75 (0.03)
	10	6.30 (0.38)	4.57 (0.26)	0.60 (0.02)

**Table A2:** Geometric dimensions of the miscanthus MGig stems.

*MC* [%]	*NrNod*	*Dmax* [mm] Mean (Std. Dev.)	*Dmin* [mm] Mean (Std. Dev.)	*THwall* [mm] Mean (Std. Dev.)
20.7	1	9.80 (0.55)	8.81 (0.08)	1.46 (0.07)
	2	8.79 (0.67)	8.52 (0.16)	1.40 (0.07)
	3	8.57 (0.68)	8.00 (0.26)	1.30 (0.06)
	4	8.46 (0.69)	7.30 (0.32)	1.25 (0.06)
	5	8.35 (0.7)	6.76 (0.43)	1.14 (0.05)
	6	7.35 (0.73)	5.74 (0.46)	1.07 (0.05)
	7	6.99 (0.73)	5.63 (0.50)	0.93 (0.04)
	8	6.79 (0.77)	5.56 (0.52)	0.85 (0.04)
	9	6.68 (0.75)	5.53 (0.57)	0.74 (0.03)
	10	6.60 (0.73)	5.52 (0.55)	0.58 (0.03)
25.6	1	9.78 (0.39)	8.83 (0.11)	1.47 (0.06)
	2	8.87 (0.45)	8.55 (0.17)	1.41 (0.06)
	3	8.63 (0.38)	8.09 (0.30)	1.31 (0.05)
	4	8.53 (0.38)	7.28 (0.28)	1.26 (0.05)
	5	8.43 (0.41)	6.85 (0.35)	1.15 (0.05)
	6	7.42 (0.42)	5.89 (0.36)	1.08 (0.04)
	7	6.99 (0.43)	5.81 (0.36)	0.94 (0.04)
	8	6.78 (0.42)	5.74 (0.36)	0.85 (0.03)
	9	6.68 (0.46)	5.73 (0.38)	0.74 (0.03)
	10	6.60 (0.40)	5.71 (0.32)	0.59 (0.02)
